# A Survey on Cannabinoid Treatment of Pediatric Epilepsy Among Neuropediatricians in Scandinavia and Germany

**DOI:** 10.3389/fped.2020.00416

**Published:** 2020-07-24

**Authors:** Claus Klingenberg, George Mouslet, Helle Hjalgrim, Thorsten Gerstner

**Affiliations:** ^1^Paediatric Research Group, Faculty of Health Sciences, University of Tromsø, Tromsø, Norway; ^2^Department of Paediatrics and Adolescent Medicine, University Hospital of North-Norway, Tromsø, Norway; ^3^Clinic for Children, Værløse, Denmark; ^4^Department of Child Neurology and Rehabilitation (HABU-A), Sørlandet Hospital, Arendal, Norway

**Keywords:** cannabinoid, refractory epilepsy, Dravet syndrome, Lennox-Gastaut syndrome, survey

## Abstract

**Objectives:** There is an increasing interest in cannabinoid-based products for the treatment of refractory pediatric epilepsy. However, a licensed cannabidiol (CBD) product was first approved for use by the European regulatory authorities in 2019. We aimed to obtain knowledge about clinical experience and attitudes toward cannabinoid use for epilepsy treatment among neuropediatricians in Scandinavia and Germany in the era before a CBD-product was commercially licensed and available.

**Study design:** An internet-based questionnaire (Survey Monkey) was distributed by email to members of neuropediatric societies in Sweden, Germany, Denmark, and Norway between February and April 2018. One reminder email was sent.

**Results:** Eighty-six responded. Only 10 of 86 (12%) respondents had personal experience with off-label prescription of cannabinoid-based products, mainly for severe refractory pediatric epilepsies like Dravet syndrome and Lennox-Gastaut syndrome. However, 49 respondents (57%) had been exposed to relatives of patients that had requested or wanted to discuss cannabinoid therapy, and 32 (37%) respondents knew about cannabinoid self-medication. The knowledge regarding cannabinoid-based therapy among the respondents was overall limited. Main reasons for not prescribing cannabinoid-based therapy were concerns about law regulations and lack of an available product.

**Conclusion:** Off-label cannabinoid-based therapy for pediatric epilepsy was not widely prescribed by neuropediatricians in Scandinavia and Germany in 2018.

## Introduction

Over the last decade there has been an increasing interest in cannabinoid-based products for the treatment of refractory pediatric epilepsy (1). In animal studies cannabidiol (CBD) shows an anticonvulsant profile largely devoid of the adverse psychoactive effects which is mainly related to the tetrahydrocannabinol compound of cannabinoids (1). Anti-seizure properties of CBD are probably mediated by modulation of intracellular calcium levels, through inhibition of the G-protein coupled receptor GPR55 and activation of the transient receptor channel TRPV1, and the inhibition of adenosine re-uptake (2). Until a few years ago most data on the use of CBD-enriched extracts to treat pediatric epilepsy had been based on observational studies, mainly from the USA.

Since 2016, three randomized placebo-controlled adjunctive-therapy trials of purified CBD-products including children and adolescents with refractory epilepsy have been published (3–5). In these trials, CBD was superior to placebo in reducing the frequency of convulsive seizures in patients with Dravet syndrome (3), and in reducing the frequency of drop-seizures in patients with Lennox-Gastaut syndrome (LGS) (4, 5).

The objective of this survey was to obtain knowledge about clinical experience and attitudes toward cannabinoid use for epilepsy treatment among neuropediatricians in Scandinavia and Germany in the era before a licensed product was available.

## Materials and Methods

Between February and April 2018 we distributed via email an internet-based survey (Survey Monkey, Portland, OR, USA) to members of neuropediatric societies in Sweden, Denmark, and Norway. We assume that around 250–300 active neuropediatricians work in Scandinavia, but the sub-specialization is not uniformly formalized and the number of members of the neuropediatric societies is therefore uncertain and may not reflect all neuropediatricians treating children with epilepsy. We also sent the survey to a random group of 140 neuropediatricians in Germany. Email addresses were obtained from society web sites or email lists, and distributed by designated persons (see acknowledgment section).

The survey consisted of 16 questions using a combination of open and fixed-response questions (Appendix in Supplementary Material). We asked specific questions around knowledge, experience and attitude regarding cannabinoid use for pediatric epilepsy. Moreover, we asked whether parents or relatives had asked for these treatments for their child, and to what extent they knew about self-medication with cannabinoids. Follow-up for non-responders was by one-1 reminder email. Data are presented as number and percentages.

## Results

Eighty-six neuropediatricians responded; 42 (49%) were females (Table 1). Fifty-six respondents (65%) had more than 10 years of clinical experience in neuropediatrics, and 57 (66%) answered that they treat children with epilepsy at least every week. Eighty-one respondents (94%) had heard about of the use of cannabinoids for treating epilepsy in children. Forty-seven (55%) knew that CBD, and not THC, is the compound of cannabinoids that is suggested to be most important for anti-epileptic activity.

**Table 1:** Response from 86 neuropaediatricians from Sweden (*n* = 26), Denmark (*n* = 17), Norway (*n* = 23), and Germany (*n* = 20) on aspects related to cannabinoid therapy for epilepsy.

**Respondents who had prescribed cannabinoid-therapy (*****n*** **=** **10)**	
**Indications** ^**x**^	***N*** **(%)**
• Lennox-Gastaut syndrome	5 (50)
• Dravet syndrome	4 (40)
• Infantile spasms	2 (20)
• Tuberous sclerosis	2 (20)
**Perceived efficacy** ^**x**^	
• Good seizure burden reduction (> 90%)	0 (0)
• Moderate seizure burden reduction (> 50%)	4 (40)
• Shortening of seizures	1 (6)
• Less “severe seizures”	4 (40)
• Better life quality	4 (40)
• Improved cognition	2 (20)
• No obvious effects	4 (40)
**Respondents who had not prescribed cannabinoid-therapy (*****n*** **=** **76)**	
**Reasons for not prescribing** ^**x**^	
• No licensed product available	25 (33)
• National law regulations prohibits prescription	14 (18)
• Skepticism toward efficacy	21 (28)
• Lack of knowledge/experience	39 (51)
• Other	22 (29)

x *Multiple answers possible*.

Only 10 respondents (12%) had personally prescribed off-label cannabinoids for treatment of children with epilepsy. These caregivers had mostly prescribed a CBD oil or another purified CBD-formulation. The indications for using cannabinoids and the perceived efficacy are shown in Table 1. We also asked for adverse effects when cannabinoids were prescribed. Lethargy/drowsiness, gastrointestinal symptoms, and decreased appetite where those most frequently reported. We did not ask for liver function abnormalities and none of the respondents spontaneously reported this side effect.

The 76 respondents who had not prescribed cannabinoids for pediatric epilepsy gave several reasons for not using this therapy (Table 1). However, 49/76 (64%) had been exposed to parents or relatives of patients that had requested or wanted to discuss cannabinoid therapy. Moreover, 32 of all 86 respondents (37%) were aware of cannabinoid self-medication, not prescribed by a doctor.

## Discussion

This is the first survey on clinical experience and attitudes toward use of cannabinoid therapy for refractory epilepsy among neuropediatricians in North-Europe. A recent, similar online survey reported data from 155 physicians treating children or adolescents for epilepsy within eight countries in Central- and South-Europe (7). In that survey 45% of the respondents prescribed off-label cannabinoids (mainly CBD) for epilepsy, but the response rate was not presented (5). Moreover, the authors reported that individual experience with cannabinoid therapy among responders was limited, and there were diverse opinions about the use of CBD and how to manage the CBD treatment (7).

The main finding of the current survey was that very few neuropediatricians in Scandinavia and Germany had prescribed off-label cannabinoid therapy for pediatric epilepsy. However, those who had prescribed this therapy followed the indications used in recent clinical trials on CBD-based therapy for refractory epilepsies (3–5).

Respondents that had not prescribed cannabinoid-based therapy gave different reasons. Concerns about law regulations and lack of an available product (off-label use) was commonly cited. This may change since a purified CBD-formulation (Epidiolex®/Epidyolex®, GW Pharma) is approved by both the American Food and Drug Administration (June 2018) and the European Medicine Agency (September 2019). Recent clinical trials support a possible adjunctive role of CBD-based therapy for selected refractory epilepsies such as Dravet syndrome and LGS, two of the most severe and difficult-to-treat forms of childhood onset epilepsy (3–5). Long-term CBD treatment has showed an acceptable safety profile and a sustained reductions in seizure frequency in patients with these two epileptic encephalopathies (2, 8–10). This was confirmed in two recently published systematic reviews on CBD therapy including nearly 800 patients (children and adults) with Dravet syndrome (2) and LGS (9). These systematic reviews concluded that adjunctive add-on CBD therapy resulted in a significant reduction in seizure frequency compared to placebo, but also in a higher rate of adverse events (AEs). Typical AEs were somnolence, sedation, decreased appetite, pneumonia, diarrhea, and increased liver enzymes. Nevertheless, the overall conclusion was that add-on CBD is an efficacious long-term treatment option for these group of patients and that CBD is well-tolerated. CBD has relatively few side effects, but interactions with other medications should be monitored carefully (2, 9, 10). It also has to be acknowledged that only a purified, pharmaceutical formulation of cannabidiol has been authorized, and data from RCTs included in these systematic reviews cannot be extrapolated to other cannabinoid products.

The lack of clinical experience, but also theoretical knowledge, with CBD therapy for epilepsy in children was striking in our survey. In other countries, and in particular in the USA, off-label use of cannabinoids has increased over the last decade (1). Before initiating CBD-therapy clinicians must be aware of the most common side effects such as gastrointestinal problems, drowsiness, and liver function abnormalities (1, 3–5, 7, 8). Moreover, it is of great importance that interactions with commonly used anti-epileptic drugs (AEDs) are appreciated by doctors prescribing CBD-based therapy (1, 5, 6, 11). It has been shown that CBD may exhibit numerous interactions with other AEDs, both enzyme inducers (such as carbamazepine or phenytoin) and inhibitors (such as valproic acid), even if the clinical significance not yet is clear. CBD may have an impact on liver metabolism with significant elevation of liver enzymes in up to 10% of treated patients, of whom more than three quarter had received concomitant valproic acid therapy. In addition, there are clinically significant interaction between CBD and clobazam, an AED widely used in patients with both Dravet and LGS. The use of CBD (by inhibition of CYP2C19) may lead to a significant increase in clobazam metabolites, leading to toxicity. At the same time, clobazam may increase an active metabolite of CBD, which could lead to better seizure control. Overall, there are still many unanswered questions regarding the impact of interactions between CBD and other AEDs. However, due to an increased risk of side effects such as somnolence and increased liver enzymes, close clinical and biochemical monitoring is recommended (6, 10, 11). Moreover, it has to be emphasized that clinicians need to report all possible severe side effects, preferably by enrolling patients in post-authorization Phase IV safety studies.

The greatest limitation with our survey, and the one published by Klotz et al. (7), is that that we do not have the exact number of neuropediatricians that received the survey. We can therefore not give an exact response rate. Certainly many neuropediatricians on our email lists may in their daily practice focus on other pediatric neurology subspecialties (e.g., congenital neuromuscular disorders, cerebral palsy, and neurohabilitation) and thus do not have the same focused interest in or experience with therapy of refractory epilepsies. However, those who responded were overall very experienced neuropediatricians and around 2/3 were treating children with epilepsy on a weekly basis. We speculate that many of those who did not respond were less interested or familiar with the topic of CBD therapy and refractory epilepsies.

In conclusion, this survey indicates that off-label cannabinoid-based therapy for pediatric epilepsy in children was not widely prescribed by neuropediatricians in Scandinavia and Germany in 2018. However, self-medication and parental request for this therapy was common. This may reflect a previously reported disparity in opinion on the use of cannabinoids in epilepsy treatment with fewer medical specialists supporting its use compared with patients and the public (12). Evidence from recent clinical trials and improved availability of an approved CBD formulation will most likely change the situation regarding CBD-based therapy for pediatric refractory epilepsy in the future.

## Data Availability Statement

The raw data supporting the conclusions of this article will be made available by the authors, without undue reservation.

## Author Contributions

CK and GM had primary responsibility for the idea and design of this survey, survey distribution in Norway, data analysis and writing of the manuscript. TG participated in the design of the survey, distribution of the survey in Germany and contributed with writing of the manuscript. HH participated in the design of the survey, distribution of the survey in Denmark and writing of the manuscript. All authors have read and approved the manuscript before submission.

## Conflict of Interest

The authors declare that the research was conducted in the absence of any commercial or financial relationships that could be construed as a potential conflict of interest.
