# Supplementary material for: A Survey on Cannabinoid Treatment of Pediatric Epilepsy Among Neuropediatricians in Scandinavia and Germany
Source: Front Pediatr. 2020 Jul 24;8:416. doi: 10.3389/fped.2020.00416 (PMC7396558; doi:10.3389/fped.2020.00416)
Supplement: Supplementary file 1 [file Data_Sheet_1.PDF]

Appendix

International survey on cannabinoid treatment of epilepsy in children

Dear Colleague

Over the last decades there has been reports on the use of cannabinoids to treat children with epilepsy. Different cannabinoid formulations has been used that contains different amounts of tetrahydrocannabinol (THC) and cannabidiol (CBD).

We are conducting this international online survey among neuropaediatricians in Norway, Sweden, Denmark, Germany. The purpose is to investigate caretakers knowledge, attitudes and experiences (or lack of) with cannabinoids for treatment of epilepsy in children and adolescents (< 18 years of age).

We greatly appreciate your participation in this short online survey, which we expect to take no more than 5 minutes to complete. Replies will be treated in confidence and results made public in summarized form only.

Thank you in advance

George Mosulet, Bjørn Bjurulf, Thorsten Gerstner, Helle Hjalgrim and Claus Klingenberg,

Tromsø, Norway
Gothenburg, Sweden
Copenhagen, Denmark
Arendal, Norway

Background data

* 1. Gender

- ◯ Male
- ◯ Female

* 2. Age

- ○ 30-39
- ○ 40-49
- ○ 50-59
- ○ 60-69
- ○ 70-79

* 3. Country where you work

- ○ Germany
- ○ Norway
- ○ Sweden
- ○ Denmark

* 4. Work experience with epilepsy

- ○ I see and treat children with epilepsy at lest every week
- ○ I see and treat children with epilepsy at lest every month
- ○ I do not regularly treat children with epilepsy
- ○ Other (please specify)

* 5. Work experience in the field of neuropaediatrics

- ○ 0-4 years
- ○ 5-9 years
- ○ 10-14 years
- ○ 15 years or more

Main survey

* 6. Have you read or heard about the use of cannabinoids for treatment of epilepsy in children?

- ◯ Yes
- ◯ No

7. Which component of cannabinoids is suggested to be most important for anti-epileptic activity

- ◯ THC
- ◯ CBD
- ◯ THC and CBD
- ◯ Do not know

* 8. Have you personally prescribed cannabinoids for treatment of children with epilepsy?

- ◯ Yes
- ◯ No

9. If you have not prescribed cannabinoids for epilepsy therapy in children - what are the reasons?

- [ ] No licenced product available
- [ ] Law regulations prohibiting the use of canabinoid (off-label)
- [ ] Skepticism towards efficacy of this therapy
- [ ] Lack of knowledge/experience with this therapy
- [ ] I have prescribed cannabinoids for treatment of epilepsy
- [ ] Other (please specify)

10. If you have prescribed cannabinoids for treatment of children with epilepsy - which product did you use?

11. If you have prescribed cannabinoids for epilepsy therapy in children - which patients did you treat?

- [ ] Dravet syndrome (DS)
- [ ] Lennox-Gastaut syndrome (LGS)
- [ ] Infantile spasms (IS)
- [ ] Tuberous sclerosis complex (TSC)
- [ ] Other severe early-onset treatment-resistant epilepsies

12. If you have prescribed cannabinoids for epilepsy therapy in children - what is your impression of clinical efficacy?

- [ ] Moderate seizure burden reduction (> 50%)
- [ ] Good seizure burden reduction (> 90%)
- [ ] Shortening of seizures
- [ ] Less severe seizures
- [ ] Better life quality
- [ ] Improvement of cognitive ability
- [ ] No obvious effects

13. If you have prescribed cannabinoids for epilepsy therapy in children - which side effects did you experience or observe?

- [ ] Lethargy and drowsiness
- [ ] Gastrointestinal symptoms e.g. diarrhoea and vomiting
- [ ] Increased convulsions
- [ ] Fever
- [ ] Decreased appetite
- [ ] Other (please specify)

14. Has any of your patients or their relatives requested cannabinoid therapy for epilepsy?

- ◯ Yes
- ◯ No

15. Are you aware of cannabinoid self-medication (not prescribed by a doctor) among your patients with epilepsy?

- ◯ No
- ◯ Yes, 1-2 patients
- ◯ Yes, 3-4 patients
- ◯ Yes, at least 5 patients

16. Have you prescribed cannabinoid therapy to children/adolescents for any other indications than epilepsy?

- ☐ Yes
- ☐ No
- ☐ If yes, please describe shortly

Thank you for participating in this survey!
